# Perinatal outcome in children born after assisted reproductive technologies

**DOI:** 10.1080/03009734.2020.1726534

**Published:** 2020-03-03

**Authors:** Ulla-Britt Wennerholm, Christina Bergh

**Affiliations:** a Department of Obstetrics and Gynaecology, Institute of Clinical Sciences, Sahlgrenska Academy, Gothenburg University, Sahlgrenska University Hospital East, Gothenburg, Sweden;; b Department of Obstetrics and Gynaecology, Institute of Clinical Sciences, Sahlgrenska Academy, Gothenburg University, Reproductive Medicine, Sahlgrenska University Hospital, Gothenburg, Sweden

**Keywords:** Assisted reproductive technology, ART, ICSI, IVF, perinatal, short-term follow-up

## Abstract

Over the past 40 years access and effectiveness of assisted reproductive technologies (ART) have increased, and to date more than 8 million children have been conceived after ART globally. Most pregnancies resulting from ART are uncomplicated and result in the birth of healthy children. Yet, it is well known that pregnancies following ART are more likely to be affected by obstetric complications such as hypertensive disorders in pregnancy, preterm birth, and low birth weight compared with spontaneously conceived pregnancies. ART children are also at increased risk of birth defects. The majority of the problems arise as a result of multiple pregnancies and can be reduced by transferring a single embryo, thereby avoiding multiple pregnancies. New ART technologies are constantly introduced, and monitoring of the health of ART children is crucial.

## Introduction

Infertility affects one in seven couples, and many of these need assisted reproductive technology (ART). To date, more than 8 million children have been conceived after ART globally (1), and up to 6% (range between 0.2% and 6.4%) of the European birth cohorts is conceived by ART (2). ART involves standard *in vitro* fertilisation (IVF) and intracytoplasmic sperm injection (ICSI).

ICSI is the more advanced method, where a single sperm is injected into the cytoplasm of the oocyte. ICSI was originally used for severe male-factor infertility, but nowadays it is also used to treat mild male-factor infertility, mixed infertility, unexplained infertility, and fertilisation failures. There has been an increasing global use of ICSI, with 71.3% of fresh IVF/ICSI cycles performed with ICSI in Europe in 2014, as shown in the latest reports from European Society of Human Reproduction and Embryology (ESHRE) (2). Among fresh IVF cycles in the United States, ICSI use increased from 36.4% in 1996 to 76.2% in 2012, with the largest relative increase among cycles without male-factor infertility (3).

Further, cryopreservation (freezing and thawing of embryos) has gained popularity. In Europe, cryopreservation constituted 27.4% of all cycles in 2014, with the highest rate in Switzerland, 41.1% (2). Elective freezing of all good-quality embryos and transfer in subsequent cycles, i.e. elective frozen embryo transfer (eFET), has recently been introduced as a way to reduce ovarian hyperstimulation syndrome and improve reproductive outcome (4).

While most births after ART are uncomplicated, ART is associated with potential adverse obstetric outcomes for both mothers and infants, including hypertensive disorders of pregnancy, preterm delivery, and low birth weight (5,6). ART has also been associated with an increased risk of birth defects (7,8). Many of these adverse outcomes can be attributed to a higher rate of multiple pregnancies after ART (2,9). With the increasing use of single embryo transfer, the multiple pregnancy rate has been significantly reduced but is still unacceptably high in many countries. In 2014, ESHRE reported a multiple birth rate of 17.5% in ART in Europe (range 4.3% to 30.6%) (2). In 2016, in the US, 31.5% of ART-conceived infants were born after multiple birth pregnancies compared with 3.4% of all infants in the general population (9). However, most data also show that ART singletons have a more compromised perinatal outcome compared to singletons born after spontaneous conception, e.g. higher rates of preterm birth and low birth weight (10). New ART technologies are continuously being introduced, and it is important to monitor the safety of ART and the health of ART offspring.

The aim of this narrative review is to give a summary of the current literature on perinatal outcomes in singletons born after ART, including IVF, ICSI, freezing/thawing, oocyte donation (OD), and more recent methods such as blastocyst culture and vitrification. The review is based on recent systematic reviews (SRs) and large cohort studies. In case of rare outcomes, cohort studies with limited numbers of children have been included. Studies on perinatal outcomes after medically assisted reproduction including intrauterine insemination, preimplantation diagnostics, preimplantation genetic testing for aneuploidy, and oocyte vitrification have been excluded.

## Perinatal outcome

### IVF/ICSI versus general population

Several SRs and large cohort studies have analysed perinatal outcomes in ART singletons versus singletons from the general population (6,11–17). Most studies have included children born after IVF and ICSI as well as fresh and frozen-thawed embryo transfer (FET). The latest SR and meta-analysis from 2017 included more than 180,000 ART singleton pregnancies from 52 cohort studies from all over the world (6).

Results from the SRs consistently show that singletons born after ART have more adverse perinatal outcomes compared with singletons born after spontaneous conception, even after adjustment for relevant confounders (Table 1). ART singletons have significantly higher rates of preterm birth, with adjusted risks in the range of 1.4–2.0 for preterm birth and 1.7–3.1 for very preterm birth.

**Table 1.** Perinatal outcome in singletons born after ART versus spontaneous conception. Results from systematic reviews and Meta-analyses.

Author, year of publication (ref.)	Helmerhorst et al., 2004 (11)	Jackson et al., 2004 (12)	McGovern et al., 2004 (13)	McDonald et al., 2009 (14)	Pandey et al., 2012 (15)	Qin et al., 2017 (6)
	RR (95% CI)	OR (95% CI)	RR (95% CI)	RR (95% CI)	RR (95% CI)	ART vs SC, %
No. of studies (no. of ART children)	14 (5361)	14 (12,283)	27 (14,748)	15 (31,032)	22 (27,819)	52 (181,741)
Preterm birth						
<37 weeks	2.04 (1.80 − 2.32)	1.95 (1.73 − 2.20)	1.98 (1.77 − 2.22)	1.84 (1.54 − 2.21)	1.54 (1.47 − 1.62)	10.9 vs 6.4*
<32 weeks	3.27 (2.03 − 5.28)	3.10 (2.00 − 4.80)	2.49 (0.86 − 7.21)	2.27 (1.73 − 2.97)	1.68 (1.48 − 1.91)	2.4 vs 1.2*
Low birth weight						
<2500 g	1.70 (1.50 − 1.92)	1.77 (1.40 − 2.22)	–	1.60 (1.29 − 1.98)	1.65 (1.56 − 1.75)	8.7 vs 5.8*
<1500 g	3.00 (2.07 − 4.36)	2.70 (2.31 − 3.14)	–	2.65 (1.83 − 3.84)	1.93 (1.72 − 2.17)	2.0 vs 1.0*
Small for gestational age	1.40 (1.15 − 1.71)	1.60 (1.25 − 2.04)	–	1.45 (1.04 − 2.00)	1.39 (1.27 − 1.53)	7.1 vs 5.7*
Perinatal mortality	1.68 (1.11 − 2.55)	2.19 (1.61 − 2.98)	–	–	1.87 (1.48 − 2.37)	1.1 vs 0.6*

*P = 0.000. ART: assisted reproductive medicine; CI: confidence interval; OR: odds ratio; RR: relative risk.

Preterm birth is a syndrome with multifactorial aetiology. ART pregnancies have more placental complications (i.e. hypertensive disorders of pregnancy, placenta previa, and placental abruption) which entail an increased risk for indicated preterm birth (18–20). An increased risk of spontaneous preterm birth (<37 weeks [10.1% versus 5.5%] and <34 weeks [3.6% versus 2.2%]) in ART versus spontaneous conception has recently been reported in a SR and meta-analysis (21).

Analogously, ART singletons have higher rates of low birth weight (adjusted risks 1.6–1.7) and very low birth weight (adjusted risks 1.8–3.0) compared with spontaneous conception (6,11–15,17) (Table 1). Most studies also show an increased risk for ART singletons being born small for gestational age (SGA), with adjusted risks around 1.5, and increased risks of perinatal mortality, with adjusted risks between 1.7 and 2.0. An Australian study found that ART singletons had a 2-fold increased risk of stillbirth (17). A large Nordic collaborative study from the Committee of Nordic ART and Safety (CoNARTaS) including 62,485 ART singletons found an increased risk for stillbirth only before 28 weeks (adjusted risk 2.0) (16).

Sibling studies where the same mother has given birth to both an ART and a spontaneously conceived singleton is a way to examine ART methods *per se* under the assumption that maternal factors (except maternal age) remain constant across pregnancies. In a meta-analysis by Pinborg et al., the risk of preterm birth in a singleton sibling born after ART was higher than in a singleton sibling born after spontaneous conception (aOR 1.27, 95% CI 1.08–1.49) (22).

*In summary*, it is well documented that ART singletons have an increased risk for preterm birth and low birth weight compared with singletons born after spontaneous conception. Both parental characteristics, such as age and underlying subfertility, and the ART technique *per se* may contribute to the more adverse outcome in ART children.

### IVF versus ICSI

When comparing ICSI with standard IVF, most large studies have found similar or lower risks of preterm birth, low birth weight, and peri/neonatal mortality in singletons born after ICSI. Pinborg et al. analysed singletons born after ICSI (fresh or frozen/thawed cycles) versus singletons born after IVF (fresh or frozen/thawed cycles) (22). Five studies were included in a meta-analysis on preterm birth. The pooled estimate for ICSI singletons versus IVF singletons showed a lower risk of preterm birth in ICSI singletons (aOR 0.80, 95% CI 0.69–0.93). A possible explanation for the better outcome in ICSI singletons may be that in ICSI the majority of the women are reproductively healthy, which could give a more favourable perinatal outcome.

*In summary*, children born after ICSI have a better perinatal outcome compared with standard IVF.

### Transfer of blastocysts versus transfer of cleavage stage embryos

Blastocyst culture (day 5–6) compared with cleavage stage culture (day 2–3) is considered to improve the selection of the most viable embryo and to increase pregnancy and live birth rates per transfer and potentially result in more healthy infants (23). Yet, SRs and meta-analyses show that the cumulative live birth rate, including a fresh transfer and all subsequent frozen embryo transfers from one oocyte retrieval, is similar for blastocyst transfer and cleavage stage transfer (23,24). However, blastocyst culture, by improving embryo selection, may encourage elective single embryo transfer and thus reduce multiple birth rates.

Two recent systematic reviews and meta-analyses on perinatal outcomes after blastocyst transfer versus cleavage transfer included more than 100,000 singletons born after fresh cycles (25,26). Both found a higher rate of preterm birth (<37 weeks; relative risks [RRs] 1.15 and 1.16, respectively) and very preterm birth (<32 weeks; RRs 1.16 and 1.16, respectively) and a lower rate of being SGA (RRs 0.83 and 0.84, respectively) after blastocyst transfer compared with cleavage stage transfer. There was no difference with regard to low birth weight.

Abnormal placentation and implantation may cause the increased risk of preterm birth after blastocyst transfer. A population-based registry study found increased rates of placenta previa and placental abruption after blastocyst transfer (27). Blastocyst transfer is associated with a higher risk for monozygotic twinning (MZT) (28–30). In a SR and meta-analysis of 38 studies, rates of MZT after blastocyst transfer varied between 0% and 13.3%, and there was a 2-fold increased risk compared with cleavage transfer (OR 2.18, 95% CI 1.93–2.48) (29). The authors suggest—besides extended culture time—culture media and the age of the mother as the underlying mechanisms (29). Further, blastocyst transfer is associated with a higher male-to-female ratio (28,31–33).

*In summary*, blastocyst transfer compared with cleavage transfer is associated with a small increased risk of adverse perinatal outcomes, particularly preterm birth. Further, a higher rate of MZT and an altered sex-ratio have been observed after blastocyst transfer.

### Fresh versus frozen/thawed embryo transfer

Several SRs and meta-analyses have observed that perinatal outcomes are better in children conceived following frozen/thawed embryo transfer (FET) compared with fresh embryo transfers, with reduced risks of preterm birth and low birth weight (22,34–36).

Maheshwari et al. performed an updated SR and meta-analysis (26 studies and almost 300,000 deliveries) and confirmed that singletons conceived from FET were at lower risk of preterm birth (RR 0.90, 95% CI 0.84–0.97), low birth weight (RR 0.72, 95% CI 0.67–0.77), and SGA (RR 0.61, 95% CI 0.56–0.67) compared with those conceived from fresh embryo transfers (37). Yet, they also found that singletons born after FET had an increased risk of being born large for gestational age (LGA) (RR 1.54, 95% CI 1.48–1.61) and being macrosomic (birth weight more than 4000 g) (RR 1.85, 95% CI 1.46–2.33). There was no difference in the risk of perinatal mortality in children born after FET versus children born after fresh embryo transfer, but the risk of hypertensive disorders of pregnancy was increased (RR 1.29, 95% CI 1.07–1.56) in pregnancies after FET.

Theories of the underlying mechanisms behind this over-growth include a selection of better embryos surviving the freezing and thawing procedure. Further, epigenetic modifications may occur at the early embryonic stages and affect the growth potential of the foetus. A third suggested theory is that the uterine environment in a FET cycle is more natural than in fresh IVF, as most FET cycles do not use the ovarian stimulation which is used in fresh IVF cycles.

Recent studies have shown a link between the absence of corpus luteum and a higher risk of pre-eclampsia (38,39). The obstetric outcome after FET depending on protocol used has recently been investigated (40). Programmed cycles (no corpus luteum, *n* = 1446) were associated with higher rates of hypertensive disorders in pregnancy, postpartum haemorrhage, postterm birth, and macrosomia compared with natural cycles (*n* = 6297). There were no differences regarding preterm birth and low birth weight. The results support the hypothesis of a link between absence of corpus luteum in programmed cycles and adverse perinatal outcomes. With the increasing number of ART cycles worldwide performed as FET, this finding is important and may support the use of natural cycles in FET.

A recent SR (11 randomised controlled studies) evaluated perinatal outcome after eFET versus fresh embryo transfers (5379 women) (4). There were higher risks of pre-eclampsia after eFET (RR 1.79, 95% CI 1.03–3.09), whereas mean birth weight, preterm birth, or birth defects were the same. Concerning safety, eFET significantly decreased the risk of moderate and severe ovarian hyperstimulation syndrome.

### Vitrification versus slow freezing

Vitrification is an ultrarapid cryopreservation method, which instead of slow freezing has become the dominant method for cryopreservation in recent years. It has been associated with higher post-thaw survival rates and higher clinical pregnancy rates when compared with slow freezing (41,42). However, the high concentrations of cryoprotectants used for vitrification have raised concerns about possible negative health effects for the children. When comparing vitrification and slow freezing of day-3 embryos (43) or blastocysts (44), similar outcomes were found. A Nordic study compared vitrified blastocyst transfers with slow freezing day-2–3 embryo transfer (45). Except for a higher risk of preterm birth in the vitrified blastocyst group (aOR 1.33, 95% CI 1.09–1.62), there were no other differences. The higher risk of preterm birth was considered to be related to extended culture.

*In summary*, the change of strategy from slow freezing to vitrification seems to be reassuring.

### Oocyte donation (OD)

The number of OD treatments has increased during recent years, and a total of 17,259 deliveries after OD was reported in 2014 from ESHRE (2), which considerably exceeds the number of deliveries reported in 2013 (+45.5%); 65% of the recipients were 40 years or older. In a recent SR including 23 studies, rates of hypertensive disorders in pregnancy (including pregnancy-induced hypertension, pre-eclampsia, severe pre-eclampsia), preterm birth, very preterm birth, low birth weight, and very low birthweight were increased after OD compared with IVF/ICSI with autologous oocytes (pooled odds ratio 2.64, 1.57, 1.80, 1.25, and 1.37, respectively) (46). The high occurrence of obstetric complications after OD has been associated with advanced maternal age. Yet, also in young women, aged <35 years, the use of donated oocytes compared with autologous oocytes was associated with a higher rate of preterm birth and low birth weight (47). In the SR by Moreno-Sepulveda et al., there was no difference in the rate of preterm birth and low birth weight when adjusted for pre-eclampsia (46). The fact that the foetus is allogenic to the mother’s immunological mechanisms may explain the higher risk of pre-eclampsia in OD pregnancies. There was a lower prevalence of pre-eclampsia in OD pregnancies when the donor was related to the recipient (48). Since OD pregnancies have nearly three times the risk of pre-eclampsia in comparison to spontaneous pregnancies, OD pregnancies should be considered as high-risk pregnancies, and single embryo transfer is highly recommended, as multiple pregnancies further add to the perinatal risks (49).

*In summary*, OD may constitute an independent risk factor for a more adverse perinatal and maternal outcome than pregnancies after ART with autologous oocytes.

## Birth defects

### ART versus general population

Large registry-based cohort studies and SRs with meta-analyses have assessed birth defects in ART singletons compared with spontaneously conceived singletons. Most studies have found an increased rate of birth defects in ART children, ranging between 30% and 70% (Table 2) (6,7,14,15,50–52). The latest review included 22 studies (40,746 ART singletons), and the point estimate for any birth defect was RR 1.41 (95% CI 1.38–1.52) (52). The risk of any birth defects (RR 1.36) and major birth defects (RR 1.41) for ART versus spontaneous conception was similar in the SR by Hansen et al. (7).

**Table 2.** Birth defects in singletons born after ART versus spontaneous conception. Results from systematic reviews and meta-analyses.

Author, year of publication (ref.)	Rimm et al., 2004 (51)	Hansen et al., 2005 (50)	McDonald et al., 2005 (14)	Pandey et al., 2012 (15)	Hansen et al., 2013 (7)	Qin et al., 2017 (6) ART vs SC, %	Zhao et al., 2020 (52)
No. of studies (no. ART children)	IVF: 8 (2064) ICSI: 6 (3948)	15 (13,059)	7 (4031)	7 (4382)	23 (48,944)	29 (77,630)	22 (40,746)
Birth defects: pooled estimates (95% CI)	IVF: 1.51 (0.85 − 2.70) ICSI: 1.33 (0.90 − 1.95)	1.31 (1.17 − 1.46)	1.41 (1.05 − 1.88)	1.67 (1.33 − 2.09)	Any: 1.36 (1.30 − 1.43) Major: 1.41 (1.33 − 1.50)	5.7 vs 3.9^a^	1.41 (1.30 − 1.52)

^a^*P* = 0.000. ART: assisted reproductive technology; CI: confidence interval; ICSI: intracytoplasmic sperm injection; IVF: *in vitro* fertilization; SC: spontaneous conception.

A Nordic cohort study from the CoNARTaS comparing ART singletons (*n* = 62,379) with singletons born after spontaneous conception (*n* = 362,215) observed an increased risk for major birth defects (3.4% versus 2.9%; aOR 1.14, 95% CI 1.08–1.20) (53). Increased rates of birth defects occurred in different organ systems: central nervous system; eye; ear, face, and neck; heart; gastrointestinal system; urinary system; and the musculo-skeletal system, with congenital heart defects being the most common defects. A SR of congenital heart defects in ART versus spontaneous conceptions (5 studies, 13,396 ART singletons) showed an increased risk of congenital heart defects in ART singletons: 1.0% versus 0.7% (OR 1.55, 95% CI 1.21–1.99) (54).

### IVF versus ICSI

Neither Lie et al. (4 studies) nor Wen et al. (24 studies) in their meta-analyses including both singletons and multiples found any increase in the risk of birth defects in ICSI compared with standard IVF (RR 1.12, 95% CI 0.97–1.28; and RR 1.05, 95% CI 0.91–1.20, respectively) (55,56). In contrast, an Australian study of 6163 ART children (singletons and multiples) found that IVF was associated with a reduced risk of any birth defect as compared with ICSI (aOR 0.68, 95% CI 0.53–0.87) (57). The risk was reduced for fresh cycles but not for frozen cycles. An overall higher rate of urogenital defects in ICSI versus IVF was found in a SR by Massaro et al. (58).

### ICSI with ejaculated versus non-ejaculated sperm

There seems to be no difference in the rate of birth defects in children conceived by ICSI using non-ejaculated sperm compared with ICSI using ejaculated sperm (59–61). The rate of major birth defects in children conceived where the fathers had non-obstructive azoospermia, obstructive azoospermia, and aspermia (*n* = 359 children) was assessed in a nationwide Norwegian study (62). Birth defects were not significantly associated with sperm origin or the cause of male-factor infertility.

### Fresh versus frozen/thawed embryo transfer

The rate of birth defects in children born after FET from slow freezing procedures seems comparable to that of ART children born after fresh cycles (34,63,64). Two recent SRs including singletons born after FET from vitrification of slow freezing found no difference in the risk of birth defects between FET and fresh cycles. (26,37). A single-centre Belgian study reported that the rate of any or major birth defects in singletons born after vitrification (*n* = 827) was similar to that after fresh cycles (*n* = 1374) (65).

### Transfer of blastocysts versus transfer of cleavage stage embryos

Earlier studies have claimed that blastocyst transfer is associated with an increased risk of birth defects compared with cleavage stage (32,66). Two recent reviews and one cohort study assessed birth defects after blastocyst transfer and found no increased risk compared with cleavage stage transfer, irrespective of whether any cryopreservation procedure had been used (25,27,67).

*In summary*, ART is associated with a modestly increased risk of birth defects, when compared with spontaneous conception. There seems to be no increased risk after cryopreservation, while the risk after ICSI is still unresolved. On an individual level the increase in birth defects is small.

## Chromosomal anomalies

Early studies form the Belgian group have shown a higher rate of *de novo*, non-inherited chromosomal abnormalities in ICSI children (*n* = 1586) compared with the rate in the general population (1.6% versus 0.5%) (68). This was related mainly to a higher number of sex chromosomal anomalies and partly to a higher number of autosomal structural anomalies. The finding was associated with sperm concentration and motility. In children born to fathers with reduced sperm concentration the incidence of *de novo* abnormalities was higher compared with children born to fathers with a normal sperm concentration (2.1% versus 0.24%) (68). The incidence of *de novo* chromosomal anomalies was comparable in children conceived from non-ejaculated sperm (testicular *n* = 530, epididymal *n* = 194) versus ejaculated sperm (*n* = 2516) (69).

*In summary*, based on few studies, ICSI may be related to a modestly increased risk of chromosomal abnormalities associated with sperm parameters.

## Trends over time

A Nordic study from the CoNARTaS group analysed trends in perinatal health in ART singletons (*n* = 62,379) versus a control group of spontaneously conceived singletons (*n* = 362,215) (70). The rates of perinatal outcomes were stratified into four time periods: 1988–1992, 1993–1997, 1998–2002, and 2003–2007. There was a substantial decline in the risk of being born preterm and very preterm for singletons conceived after ART but not for singletons born after spontaneous conception. Rates of low birth weight, stillbirth, and infant deaths also declined among ART singletons. A possible explanation for the positive development may be a change in the ART population, with healthier women with shorter time of infertility undergoing ART treatment. Other factors are increased use of ICSI for male-factor infertility, cryopreservation, and single embryo transfer. Single embryo transfer reduces the risk of the vanishing twin phenomenon, a risk factor for preterm birth (71).

Another study from the CoNARTaS group assessed the risk of major birth defects and the risk over time between 1988 and 2007 in ART singletons compared with spontaneously conceived singletons (53). The rate of children born with a major birth defect increased in both groups over time, but the difference in risk of a major birth defect between ART children and spontaneously conceived children remained unchanged.

*In summary*, singletons born after ART have a higher risk for adverse perinatal outcomes compared with singletons born after spontaneous conception. There is a positive trend with improved outcomes, mainly for rates of preterm birth during recent years.

## Comments

The conclusion from numerous studies is that ART is a safe and successful treatment for infertility. Further, perinatal outcomes have improved over time. The increased use of single embryo transfer (SET), thus avoiding multiple pregnancies, is the main contributor to the better outcome seen during recent years. Nordic countries, Sweden in particular, have been the leading countries in reduction of multiple pregnancies by implementing SET as the main strategy (72). Several studies have shown that perinatal outcome is better in ART singletons compared with ART multiples including twins (73–75). Yet, there is a modestly increased risk of adverse perinatal outcomes including birth defects in ART singletons compared with the general population. Whether this is attributable to patient characteristics related to infertility or the ART technique is uncertain. ART children have mostly been compared with children in the general population born after spontaneous conception. Some studies, however, included only spontaneous conceptions in a subfertile population as controls, while others have excluded the subfertile population. Subfertility with or without assisted conception is found to be significantly associated with a higher rate of adverse perinatal outcomes and increased risk of birth defects (22,57,76). Patients with infertility may be older and more likely to have pre-existing comorbidity, which may predispose to adverse perinatal outcomes. Most outcome data come from retrospective observational studies with a wide heterogeneity and differences in control groups, and, as in all observational studies, there may be unknown and unmeasured confounders.

There are several pitfalls and methodological limitations when birth defects are studied. The definition of birth defects, mode and frequency of pre- and postnatal surveillance, length of follow-up, inclusion or exclusion of pregnancy terminations for birth defects, source of data, etc. may differ between ART pregnancies and spontaneous conception. Most early studies included small sample sizes with inadequate power to assess differences in rates of birth defects.

The increasing and often unnecessary use of ICSI worldwide is a matter of concern, as there are still conflicting results concerning the risk of birth defects. Therefore, until further research can demonstrate safety, ICSI should mainly be reserved for its original intended use, male-factor infertility.

Caution should be applied about embarking on a policy of electively freezing all embryos in ART as there seems to be increased risks for hypertensive disorders of pregnancy, macrosomia, and LGA babies after FET. The implications of the findings of macrosomia and LGA after FET, and the consequences for future health and risk of obesity in offspring, are unclear. Therefore, eFET should be used in specific cases such as high risk of ovarian hyperstimulation syndrome, fertility preservation, or in the context of randomised trials.

Concerning management during pregnancy, closer surveillance during pregnancy and prophylactic treatment for pre-eclampsia with low-dose aspirin may be indicated in high-risk pregnancies such as pregnancies after OD (77). The higher risk of spontaneous preterm birth may indicate a benefit of screening with transvaginal ultrasound measurements of cervical length in the second trimester and subsequent treatment with progesterone if the cervix is short (78).

Continuous supervision after ART is needed to ensure safety and quality, especially when new techniques are introduced. National ART registries such as those existing in the Nordic countries enable follow-up studies of ART children and should be encouraged. Furthermore, research collaborations between countries should be supported as well (79).
